# Design and Numerical Analysis of an Inside-Beam Powder Feeding Nozzle for Wide-Band Laser Cladding

**DOI:** 10.3390/ma17010012

**Published:** 2023-12-19

**Authors:** Lin Lu, Tuo Shi, Gang Li, Chao Wei, Geyan Fu

**Affiliations:** 1 Department of Mechanical and Electrical Engineering, Suzhou Vocational Institute of Industrial Technology, Suzhou 215104, China; claire823@163.com; 2 School of Mechanical and Electrical Engineering, Soochow University, Suzhou 215021, China; 3 School of Optoelectronic Science and Engineering, Soochow University, Suzhou 215021, China; 4 School of Mechanical Engineering, Nanjing University of Science and Technology, Nanjing 210094, China

**Keywords:** inside-beam powder feeding, laser internal powder feeding, wide-band laser cladding, powder-feeding device

## Abstract

Wide-band laser cladding technology has emerged as a solution to the limitations of traditional cladding techniques, which are small single-path dimensions and low processing efficiency. The existing wide-band cladding technology presents challenges related to the high precision required for the laser–powder coupling and the significant powder-divergence phenomenon. Based on the inside-beam powder-feeding technology, a wide-band powder-feeding nozzle was designed using the multi-channel powder flow shaping method. The size of the powder spot obtained at the processing location can reach 40 mm × 3 mm. A computational fluid dynamics analysis using the FLUENT software was conducted to investigate the impact of the nozzle’s structural parameters on the powder distribution. It was determined that the optimal configuration was achieved when the powder-feeding channel was 8, and the transverse and longitudinal dimensions for the collimating gas outlet were 0.5 mm and 1 mm, respectively. Among the process parameters, an increase in the carrier gas flow rate was found to effectively enhance the stability of powder transportation. However, the powder feed rate had minimal impact on the powder concentration distribution, and the collimating gas flow rate appeared to have a minimal effect on the divergence angle of the powder stream. Wide-band laser cladding experiments were conducted using the designed powder-feeding nozzle, and a single-path cladding with a width of 39.96 mm was finally obtained.

## 1. Introduction

Laser cladding technology is known for its low dilution, strong metallurgical bonding of cladding layers with substrates, and fine-grained, dense microstructures. It has been used in the modification and maintenance of mechanical parts to improve their surface properties, such as corrosion resistance and durability, etc. [1,2,3,4]. Many researchers have focused on how to improve the efficiency of laser cladding, reduce the production cost, and promote its industrialization at present. High-speed laser cladding (HSLC) received great attention in recent years [5,6,7]. Because of the very high deposition speed, the technology is mainly applicable to rotary parts. Compared with HSLC, wide-band laser cladding improves the cladding efficiency by increasing the spot size to increase the scanning area per unit time, which can reduce cladding overlap defects due to fewer paths [8], and it is available for a wider range of parts. Meanwhile, compared to traditional laser cladding techniques, wide-band laser cladding technology has many advantages, such as a larger high-temperature zone width, a more uniform temperature distribution, low crack sensitivity, a uniform alloying element distribution, and a small heat-affected zone [9,10]. Consequently, this technology holds significant importance in applications involving large axial and planar components.

The current wide-band laser cladding method typically involves a solid wide-band laser beam onto the work-piece surface directly with the powder stream feeding from one or both sides [11,12,13,14,15,16,17]. This powder-feeding method necessitates high precision in the coupling of the laser and powder and is associated with substantial powder-stream divergence [18]. Lei et al. [19,20] developed a wide-band feeding nozzle and placed it inside the laser cladding head based on the inside-beam powder-feeding technology. This device enabled the vertical injection of metal powders into the laser molten pool, thereby addressing the issue of high sensitivity of the powder–laser coupling. However, a problem with the existing wide-band cladding methods is that the width of the single cladding layer is mostly less than 25 mm, and the efficiency improvement is limited [11,12,13,14,15,16,17,19,20].

On the premise of meeting the performance requirements, the larger the width of the single cladding layer, the more obvious the efficiency improvement. Building upon the inside-beam powder-feeding technology, this study employed a multi-channel powder-flow shaping method to design a wide-band laser cladding nozzle with 40 mm in the length direction, effectively increasing the size of the powder stream. Utilizing the FLUENT software (Ansys Fluent 2020R2), a simulation analysis was conducted to explore the powder distribution concentration and divergence angles under various structural parameters. A new type of wide-band laser cladding nozzle was manufactured, and a good effect of powder delivery was obtained. Compared with common wide-band cladding, the width of the single cladding layer was obviously increased, which is a benefit that improves the cladding efficiency.

## 2. Design of the Nozzle Structure and Establishment of the Simulation Model

**Figure 1:**
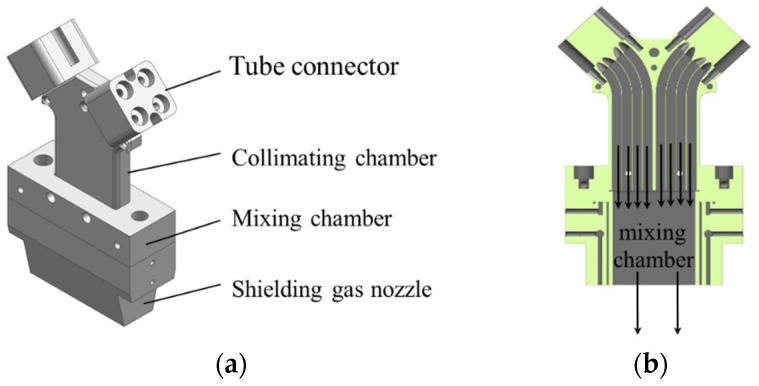
Schematic diagram of an inside-beam powder feeding nozzle for wide-band laser cladding. (**a**) Nozzle structure diagram; (**b**) Internal structure diagram.

The wide-band laser cladding powder-feeding nozzle designed in this study is shown in Figure 1. The powder was connected to the wide-band feeding nozzle through a tube connector after transport from the powder feeder. Upon entry into the nozzle, the transverse velocity of the powder stream was attenuated within the collimating chamber, thereby achieving the effects of powder collimation and stable transportation. Subsequently, the collimated multiple powder streams entered a mixing chamber where the powder mixed and filled the chamber under the influence of a high-pressure powder carrier gas. Due to the constraining effect of the chamber’s inner walls, the powder spot finally showed a rectangular shape, and the divergence was inhibited. An additional shielding gas nozzle was set surrounding the mixing chamber. During the powder-feeding process, this shielding nozzle conveyed a high-pressure, high-velocity gas flow, which provided further collimating and constraining effects as the powder flowed from the mixing chamber. Simultaneously, the shielding gas created a localized inert environment in the vicinity of the molten pool, reducing oxidation during the cladding process.

### 2.1. Model Building and Condition Setting

**Figure 2:**
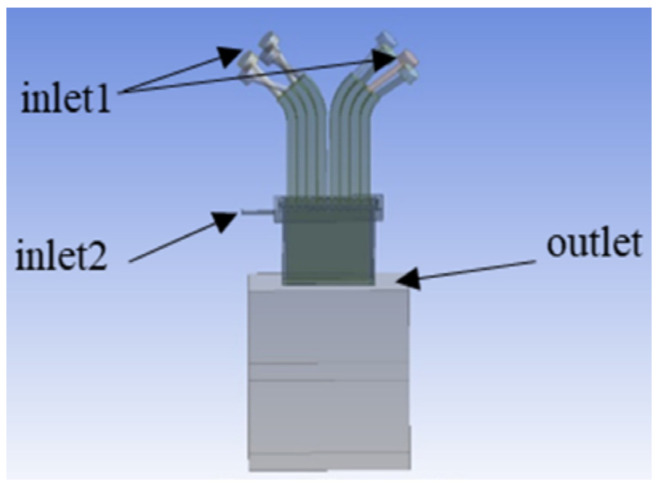
Nozzle runner model diagram.

Based on the three-dimensional model of the powder-feeding nozzle, the internal flow channels of the nozzle were extracted in the pre-processing DesignModeler of Ansys Workbench 2020R2. A 100 mm × 50 mm × 100 mm air domain was established to simulate the flow of powder after it exited the nozzle, as illustrated in Figure 2. Inlet 1 served as the entrance for the carrier gas and powder with a diameter of 9.7 mm, while Inlet 2 represented the entrance for the collimating gas with a diameter of 3 mm. Both inlets were vertical to the cross-section of the channel and configured as velocity inlet conditions. The gas was assumed to be nitrogen. The total flow rate of the carrier gas was 15 L/min, and considering the presence of 8 inlets, the velocity at Inlet 1 was calculated to be 0.42 m/s. The outlet boundary was configured as a pressure outlet condition. Other surfaces within the model were set as wall boundary conditions. Meshing of the model was performed using the Mesh module, employing a hexahedral meshing approach with a cell size of 1 mm. Furthermore, for the cylindrical sections at the inlets, such as the carrier gas and collimating gas inlets, an inflation layer mesh refinement was utilized to achieve finer mesh sizing.

**Figure 3:**
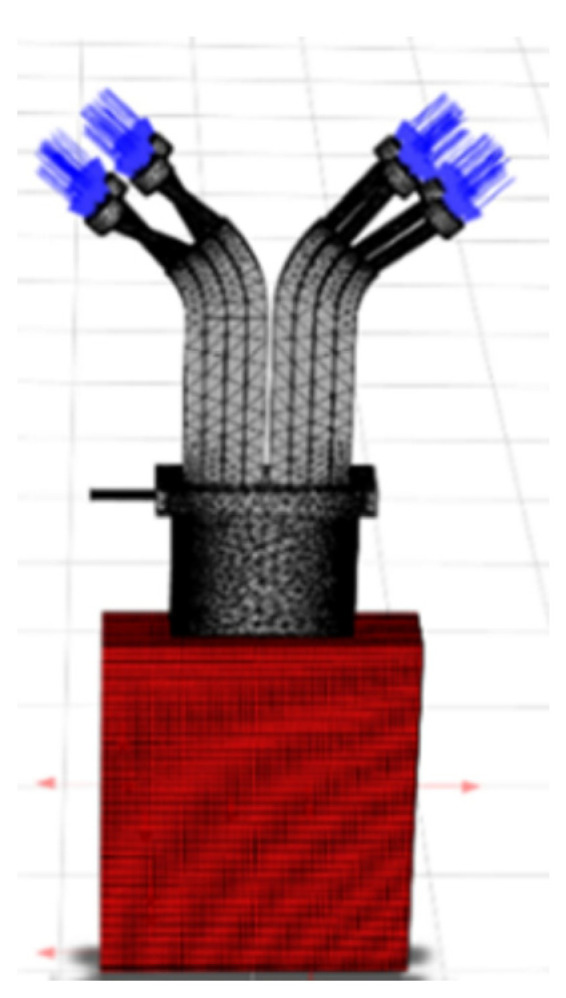
Powder–gas flow field diagram.

In order to simulate the wide-band powder and gas flow field, Ansys Fluent 2020R2 was employed, and the model is illustrated in Figure 3. The blue regions represent the inlets, and the red regions represent the outlets. Given that the volume of the powder particles relative to the carrier gas can be neglected, a two-phase flow model, specifically the gas–solid two-phase flow model, was chosen to simulate the gas flow and the powder stream with the nozzle. The carrier gas and collimating gas were treated as the continuous phase, and the SST k-omega model was selected. The powder particles were considered as the discrete phase. The injection source is linked to Inlet 1, which served as the entrance for the carrier gas. The material used for the powder was steel, and the particle size distribution followed a normal distribution ranging from 53 μm to 150 μm. The powder feed rate was set at 43.2 g/min. The simulation process involved conducting an initial simulation of the gas flow field in isolation. Once the gas flow field achieved a stable state, the discrete phase model was introduced with the consideration of the interactions between the gas and the particles.

### 2.2. Evaluation Method of Powder Distribution

The distance between the nozzle outlet and the processing plane was set at greater than 35 mm to ensure the normal temperature of the cladding nozzle, since the laser power was as high as 6 kW. To minimize the occurrence of un-melted and under-melted powder particles in the vicinity of the melt pool, it is ideal for the laser spot size to be slightly larger than the powder spot size. In the case of wide-band laser cladding, the optimal powder spot shape is a uniform rectangular distribution, matching the size of the laser spot. In the design of the wide-band cladding nozzle, the laser spot size at the processing location was specified as 40 mm × (2.5~3.5) mm. Consequently, the outlet size of the powder feeding nozzle was designed to be 40 mm × 3 mm to align with this requirement.

**Figure 4:**
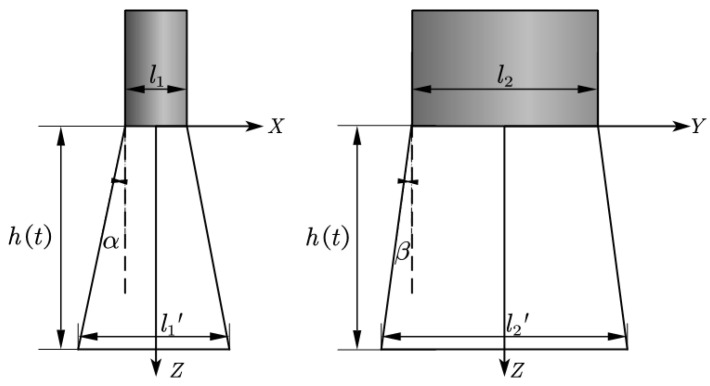
Diagram of powder divergence angle.

The assessment of the powder distribution status was conducted by evaluating the uniformity within the powder spot and examining the numerical values of the powder beam divergence angles. This study aimed to compare the impact of variations in key parameters on powder convergence behavior. Given that the powder beam from the wide-band nozzle exhibited a rectangular character, the convergence of the powder was evaluated by considering the divergence angles of the powder in the X and Y directions, denoted as α and β, respectively. These divergence angles are defined as illustrated in Figure 4.

The calculation of the divergence angles are as follows:

$$l_{1}{}^{\prime }=2h\left(t\right)\tan\alpha +l_{1}\;l_{2}{}^{\prime }=2h\left(t\right)\tan\beta +l_{2}\;\alpha =\tan^{-1}\frac{l_{1}{}^{\prime }-l_{1}}{2h\left(t\right)}\;\beta =\tan^{-1}\frac{l_{2}{}^{\prime }-l_{2}}{2h\left(t\right)}$$

where $l_{1}$ and $l_{2}$ represent the dimensions of the powder beam in the X and Y directions at the outlet, which are equal to the width and length of the nozzle, respectively. h(t) is the distance from the powder outlet to the substrate surface. $l_{1}{}^{\prime }$ and $l_{2}{}^{\prime }$ represent the dimensions of powder distribution in the X and Y directions at a distance of h(t) from the outlet, which can be obtained from the simulation results. α and β are the divergence angles of the powder stream in the XOZ and YOZ planes, respectively.

In the analysis, $l_{1}{}^{\prime }$, $l_{2}{}^{\prime }$ represented the dimensions of the powder distribution at a distance of 100 mm from the outlet in the X and Y directions, respectively. The specific parameters h(t) for the cladding process were selected based on the actual processing conditions.

## 3. Results and Discussion

The main factors affecting powder distribution are structural parameters and process parameters. The structural parameters mainly include the number of channels in the powder-feeding nozzle and the outlet size of the collimated air passage, while the process parameters mainly include the powder-feeding capacity, powder carrier gas flow, and collimating gas flow.

### 3.1. Effect of the Number of Feeding Channels on Powder Distribution

In the wide-band cladding process, the powder is transported by high-pressure N_2_ in the powder feeder through a 3 mm hose to the cladding nozzle. In order to obtain the appropriate powder beam to match the 40 mm × 3 mm laser spot size, it is necessary to split the powder flow and then mix so that it has a better uniformity when it is removed from the powder-feeding nozzle. When the collimating gas flow rate is 0 (off), the powder carrier gas flow rate is 15 L/min, and the powder feed rate is 43.2 g/min; the powder distribution state is simulated and analyzed when the number of powder-feeding channels is one (no diverting), four, and eight, respectively.

**Figure 5:**
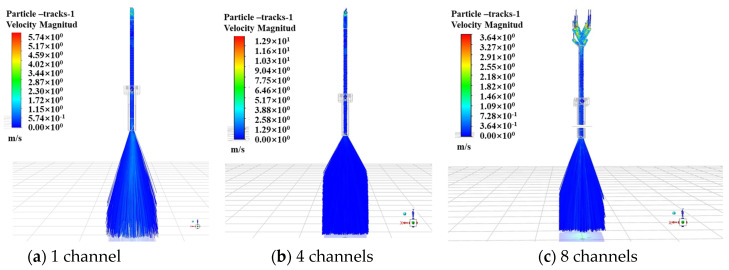
The state of the powder bundle under different numbers of powder-feeding channels (X direction).

**Figure 6:**
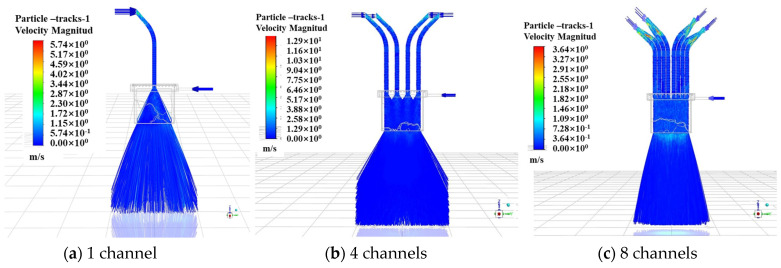
The state of the powder bundle under different numbers of powder-feeding channels (Y direction).

The status of the powder bundle under different numbers of powder-feeding channels in the X and Y directions is shown in Figure 5 and Figure 6. It can be observed that, after the powder beams fly away from the different powder-feeding channels, they enter the rectangular powder-mixing chamber. Powder particles eject and mix inside the mixing chamber and then disintegrate from the bottom of the chamber. As can be observed in Figure 6a, in the Y direction after the powder flow enters the powder-mixing chamber, it presents a certain degree of divergence, but it cannot fill the chamber in time and only shows a small amount of contact at the bottom of the mixing chamber. Because the divergence angle is limited and the diameter of the powder tube is limited by the powder-feeding hose and powder feeder, a single powder tube cannot achieve sufficient powder mixing in the central position; therefore, it is necessary to set several powder tubes that are evenly distributed above the mixing chamber to effectively increase the powder distribution range. When the powder-feeding channel is four, the powder begins to contact the inner wall of the powder-mixing chamber after entering a certain length and mixing, while the powder begins to mix and completely fills the inner space of it at the moment the powder enters the mixing chamber when there are eight channels. So, when the number of channels is eight, the uniformity and convergence of the powder bundle are the best.

**Figure 7:**
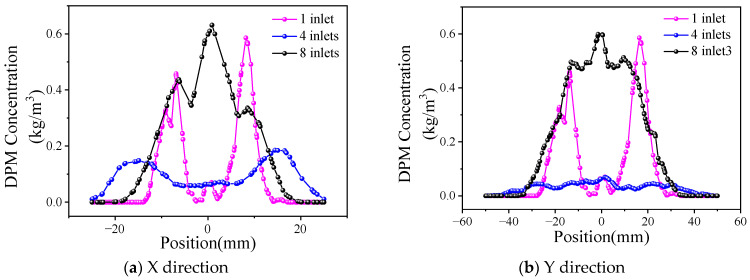
Distribution of powder concentration under different feeding channels.

The powder concentration distribution curves under different numbers of powder-feeding channels are shown in Figure 7, and the distributions in the X and Y directions have similar characteristics. Due to the small size (3 mm) in the X direction of the mixing chamber, the divergence of the powder beam is limited by the inner wall of the chamber. Meanwhile, the ejection of the powder causes the concentration of the powder beam to spread around, which results in a trend that is higher on the sides and lower in the middle of the curve. As shown in Figure 7a, when the number of channels is one, the powder concentration distribution is extremely uneven due to insufficient powder mixing. When there are four channels, the concentration distribution of the powder begins to converge to the middle when the powder enters the mixing chamber to a certain depth, but the powder state in the chamber is more chaotic; therefore, the distribution width of the powder is larger, and the divergence is more serious. In contrast, when the channel number is eight, the powder has a relatively uniform concentration distribution when it is removed from the bottom of the mixing chamber due to the sufficient ejection of the powder. The powder concentration at the center has the largest value, approximately 0.6 kg/m^3^. In the Y direction, as shown in Figure 7b, the powder concentration distribution in the case of eight inlets is relatively uniform, all of which are approximately 0.5 kg/m^3^, within the coordinate range of −20 mm~20 mm.

The powder presents Gaussian distribution in the X direction when the powder feeding channel is eight, corresponding to the energy distribution of the laser beam in this direction. At the same time, the powder is concentrated in the Y direction within ±20 mm from the center, which is the same as the design width of 40 mm in the laser beam, and can meet the coupling requirements of laser and powder better. The number of powder-feeding channels is finally determined to be eight, which is achieved by dividing the single beam of the powder tube.

### 3.2. Effect of Collimating Gas Outlet Size on Powder Distribution

**Figure 8:**
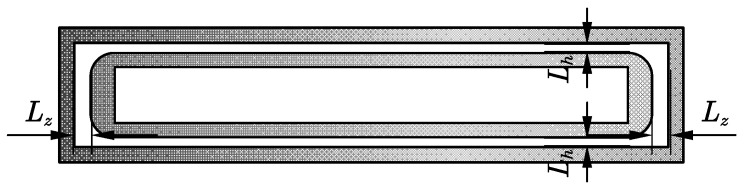
Diagram of collimating gas outlet size.

**Figure 9:**
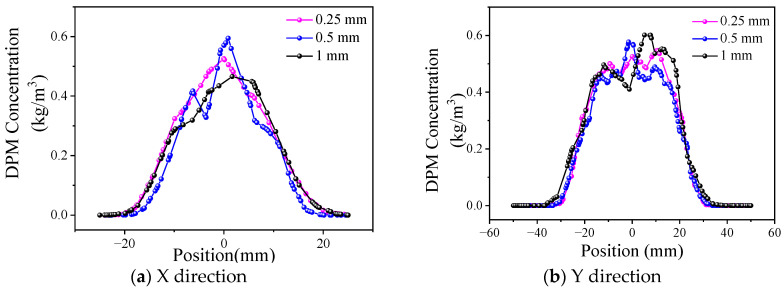
Distribution of powder concentration under different L_h_.

**Figure 10:**
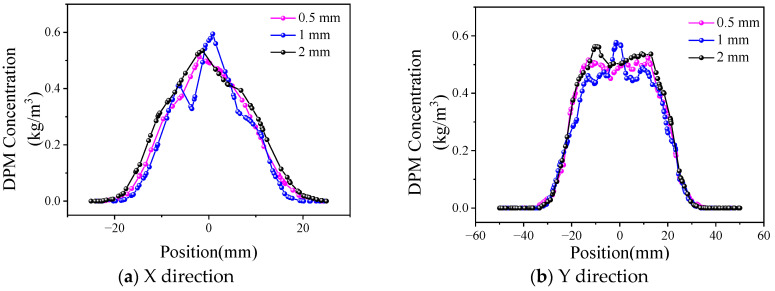
Distribution of powder concentration under different L_z_.

Collimating gas is set around the powder delivery channel, which can reduce the dispersion tendency of the powder beam and improve the bunching property of the powder. Since the powder-feeding nozzle of the wide-band cladding nozzle is rectangular, the collimating air channel is set as a coaxial rectangular ring, as shown in Figure 8. Under the same collimating gas flow rate, an outlet size that is too small will lead to a larger gas flow rate, which may disturb the central powder flow, while if the outlet size is too large, the collimating gas flow rate is small, and the effect on powder bunching is reduced. The characteristic dimensions of the collimating gas outlet are divided into transverse (L_h_) and longitudinal (L_z_). When L_z_ is fixed at 1 mm, and L_h_ is 0.25 mm, 0.5 mm, and 1 mm, respectively, the powder concentration distribution is as shown in Figure 9. Similarly, when L_h_ is fixed at 0.5 mm, and L_z_ is 0.5 mm, 1 mm, and 2 mm, respectively, the curve is as shown in Figure 10.

Figure 9 shows that the powder distribution presents characteristics similar to Gaussian distribution in the X direction. Because the outlet size of the mixing chamber in the X direction is only 3 mm and the transverse outlet size of the collimated gas is 0.25 mm to 1 mm, the collimated gas has a significant constraint effect on the powder bundle in this direction; therefore, the powder bundle is concentrated in the central region. In the Y direction, the distribution is more uniform within ±20 mm from the powder center. Because the outlet size of the mixing chamber reaches 40 mm in the Y direction and the longitudinal outlet size of the collimated gas ranges from 0.5 mm to 2 mm, the restraining effect of the collimated gas on the powder bundle is only reflected on both sides of the powder bundle; therefore, the powder concentration is a flat-top distribution.

By comparing the influence of the transverse and longitudinal collimated gas outlet sizes on the powder concentration distribution in Figure 9 and Figure 10, it can be observed that the collimated gas outlet sizes have little influence on powder distribution.

**Figure 11:**
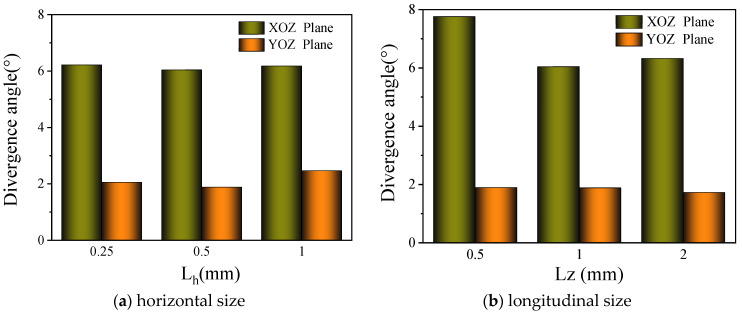
Effect of collimating gas outlet size on divergence angle.

The influence of different collimating gas outlet sizes on the divergence angle of powder in the X and Y directions is compared. In the simulation results, the powder concentration value starts from 0, while in the test process, the powder can be observed and has practical significance only when it reaches a certain concentration in the cladding process. In order to effectively calculate the dispersion angle of the powder beam, the powder distribution width d, that is the powder concentration range, needs to be determined. According to the analysis of the preliminary test data, the powder distribution range with a powder concentration ≥0.2 kg/m^3^ was defined as d. The dispersion angles of the powder beam in the X and Y directions under different collimated gas outlet sizes are shown in Figure 11.

As shown in Figure 11a, an increase in the transverse size results in more collimated gas flow out through the transverse outlet, and the flow and velocity of the longitudinal collimated gas decrease to a certain extent. When the L_h_ is increased from 0.25 mm to 0.5 mm, the gas flow rate can still provide a good collimation effect; meanwhile, due to the increase in the thickness of the gas curtain, the collimation effect is slightly improved, which results in a small decrease in the divergence angle in the XOZ plane. When the size continues to increase to 1 mm, the collimating gas flow rate further decreases, which reduces the restraining effect of the air flow and increases the divergence angle. On the whole, the change in L_h_ has just a small influence on the divergence angle of the XOZ plane, which remains at approximately 6.2°. Due to the decline of the collimating gas flow rate, the collimating effect on the intermediate powder beam decreases to a certain extent, which results in an increase in the divergence angle of the YOZ plane from 1.88° under an L_h_ size of 0.5 mm to 2.46°. In Figure 11b, the longitudinal dimensions have little influence on the divergence angle of the YOZ plane, which is within the range of 1.72~1.89°. When L_z_ is 1 mm, the XOZ plane has the smallest divergence angle of 6.04°. The effect of L_z_ on the divergence angle of the XOZ plane is similar to that of L_h_ on the YOZ plane.

L_h_ and L_z_ mainly affect the divergence angle of the YOZ and XOZ planes, respectively. When L_h_ is 0.5 mm and L_z_ is 1 mm, the best values of the divergence angle of the YOZ and XOZ planes are obtained, which are 1.88° and 6.04°, respectively. Therefore, the collimating gas outlet size is finally determined to be 0.5 mm and 1 mm in the transverse and longitudinal directions for later research.

### 3.3. Effect of Carrier Gas Flow Rate on Powder Distribution

**Figure 12:**
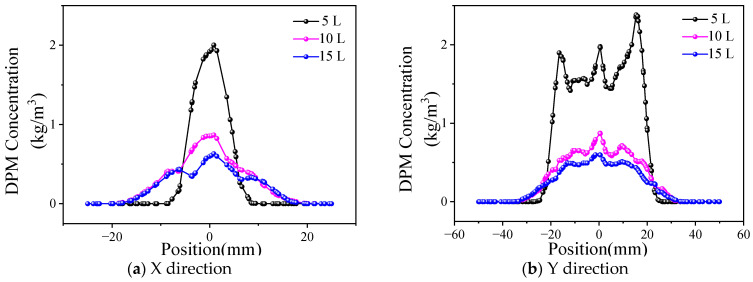
Distribution of powder concentration under different carrier gas flow rates.

The structural parameters of the nozzle were chosen in Section 2.1 and Section 2.2. When the powder feed rate is 43.2 g/min and the collimating gas flow rate is zero, the powder concentration distribution results under different powder carrier gas flow rates are shown in Figure 12. As can be observed in Figure 12a, the powder concentration distribution curve in the X direction presents characteristics similar to Gaussian distribution. When the powder carrier gas flow rate is 5 L/min, the powder obviously converges from both sides to the center, and the powder concentration at the center is as high as 2 kg/m^3^. With the same parameters in the Y direction (Figure 12b), the powder concentration within the range of −20 mm to 20 mm is generally higher, and the powder convergence is better. However, the powder concentration within the range fluctuates as high as 1 kg/m^3^, which is easy to form surface defects of uneven thickness or convex and uneven in the cladding process. With an increase in the carrier gas flow rate, the distribution range of the powder increases, and the distribution in the effective range becomes more uniform.

**Figure 13:**
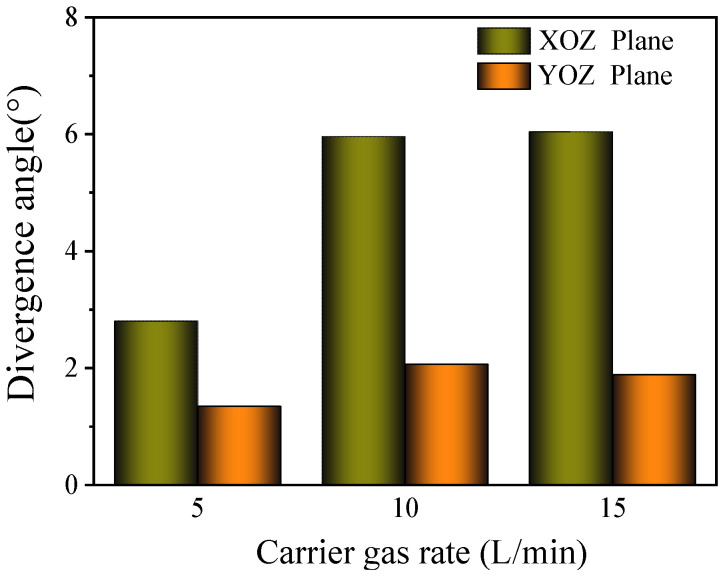
Effect of carrier gas flow rate on divergence angle.

The influence of the carrier gas flow rate on the divergence angle is shown in Figure 13. On the XOZ plane, the dispersion angle of the powder bundle is relatively stable, approximately 6°, when the flow rate of the carrier gas exceeds 10 L/min. When the flow rate of the carrier gas is 5 L/min, the divergence angle is reduced to 2.8° due to the large reduction in the flow rate and pressure of the carrier gas at the outlet of the feeder nozzle. On the YOZ plane, the divergence angle presents the same variation, and the value is 1.34°.

When the carrier gas flow rate is lower (5 L/min), the powder concentration distribution is more concentrated, and the powder divergence angles in the XOZ and YOZ directions are small. When the flow rate of the carrier gas is greater than 10 L/min, the influence of it on the powder distribution becomes smaller. In the process of the experiment, a carrier gas flow rate that is too small may lead to powder blockage in the delivery tube; increasing the parameter can effectively improve the stability of the powder transportation. Therefore, it is necessary to choose the value of the appropriate powder carrier gas flow rate according to the comprehensive consideration of powder divergence and powder transportation uniformity in the experiment.

### 3.4. Effect of Powder Feed Rate on Powder Distribution

**Figure 14:**
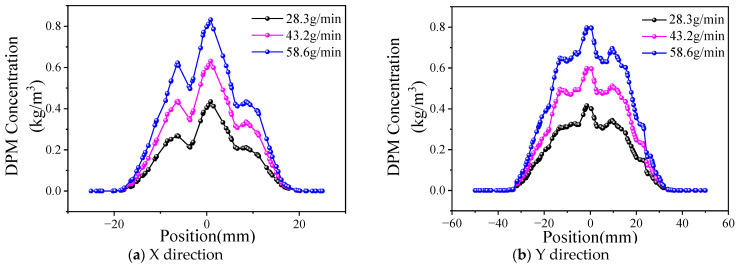
Distribution of powder concentration under different powder feed rates.

The powder concentration distribution of different powder feed rates is shown in Figure 14 (the powder gas flow rate is 15 L/min). As can be observed in the diagram, the curve shape of the powder concentration shows a consistent change in the X and Y directions when the powder feed rate increases from 28.3 g/min to 58.6 g/min. An increasing trend only appears in the height, which indicates that, when the carrier gas flow rate is fixed, the powder bunching property is basically unchanged.

**Figure 15:**
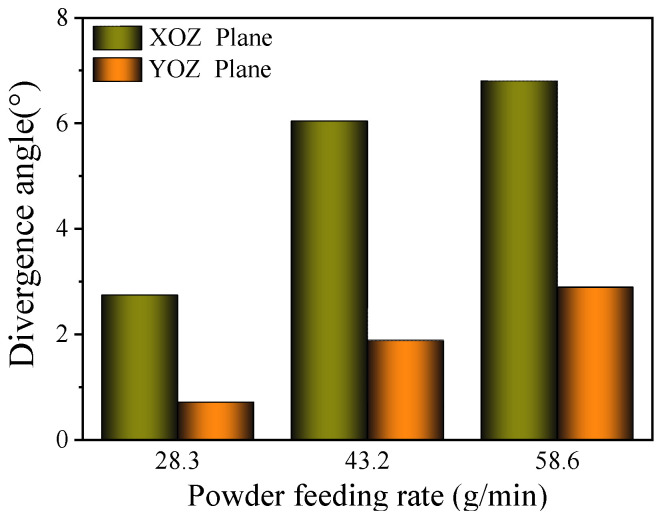
Effect of powder feed rate on divergence angle.

The influence of the powder feed rate on the powder beam dispersion angle is shown in Figure 15. When the powder feed rate increases from 28.3 g/min to 58.6 g/min, the divergence angle increases from 2.74° to 6.8° in the XOZ plane and increases from 0.71° to 2.89° in the YOZ plane. According to the conclusion of Figure 14, the bunching property of the powder does not change.

Taking into account the definition and calculation method of the divergence angle, the powder concentration in the powder beam increases with an increase in the powder feed rate; therefore, the effective distribution width of the powder with the same powder bunching property increases, resulting in an increase in the divergence angle. Therefore, the powder concentration distribution is almost not affected by increasing the powder feed rate, and the divergence angle changes with the change in the powder effective concentration region.

### 3.5. Effect of Collimating Gas Flow Rate on Powder Distribution

**Figure 16:**
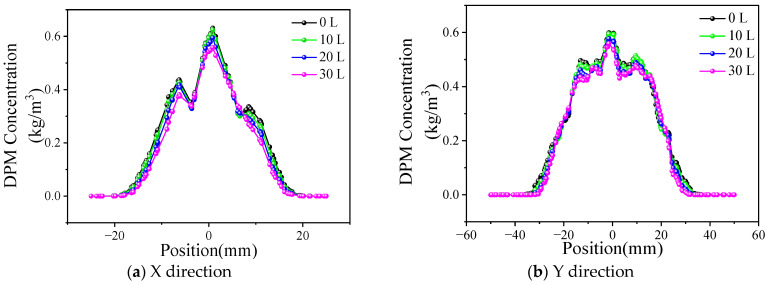
Distribution of powder concentration under different collimating gas flow rates.

The powder concentration distribution of different collimated gas flow rates is shown in Figure 16, where the powder feed rate is 43.2 g/min and the powder gas flow rate is 15 L/min.

It shows that, when the collimating gas flow rate increases from 10 L/min to 30 L/min, the powder concentration distribution curve does not change significantly compared with that when the collimating gas is closed. The collimating gas outlet area is 48 mm^2^, and the small collimating gas flow rate cannot restrain the powder bunching state too much.

**Figure 17:**
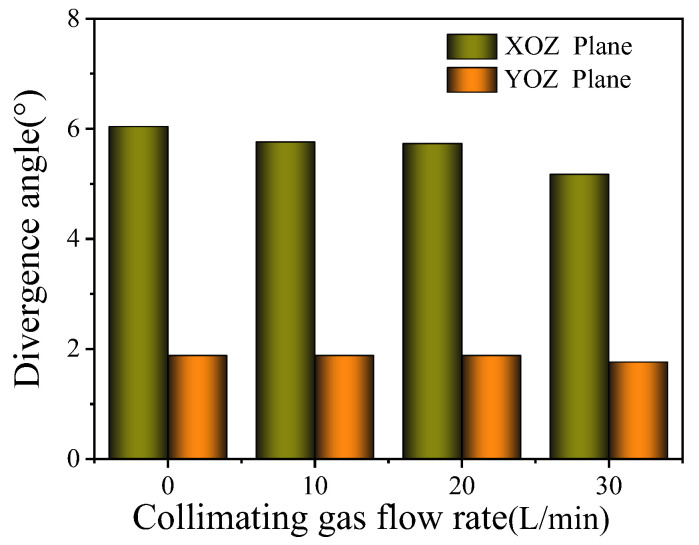
Effect of collimating gas flow rate on divergence angle.

The influence of the collimating gas flow rate on the powder beam dispersion angle is shown in Figure 17. On the YOZ plane, the divergence angle is 1.88° when the collimated gas flow rate changes from 0 to 20 L/min. When the collimated gas flow rate further increases to 30 L/min, the divergence angle decreases to 1.76°. The powder bunching property is good, so the effect of the collimating gas flow is not obvious.

## 4. Experimental Procedure

**Figure 18:**
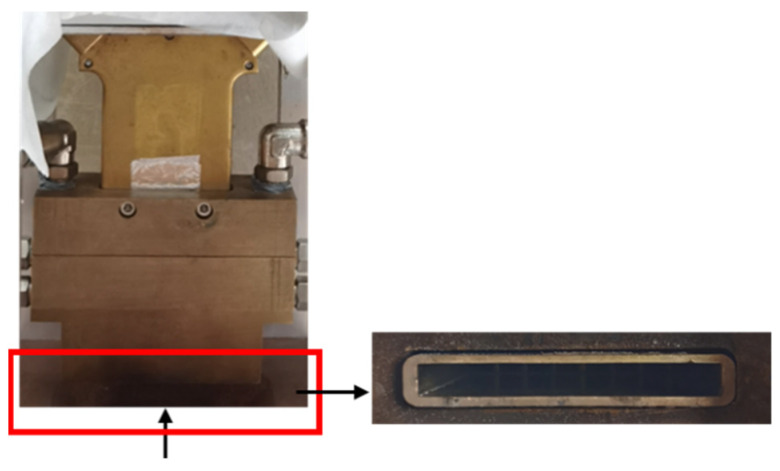
Profile of powder-feeding nozzle.

According to the numerical simulation results, it is determined that the number of powder-feeding channels is eight, the transverse outlet size of the collimating gas channel is 0.5 mm, and the longitudinal outlet size is 1 mm. The actual powder-feeding nozzle made is shown in Figure 18.

In this experiment, the PF 2/2 program controlled powder feeder (GTV, Luckenbach, Germany) was used, and high-purity N_2_ was adopted as the powder carrier gas. KF-JG-3 iron base self-soluble alloy powder was selected as the experimental material, and the particle size was 53~150 μm.

**Figure 19:**
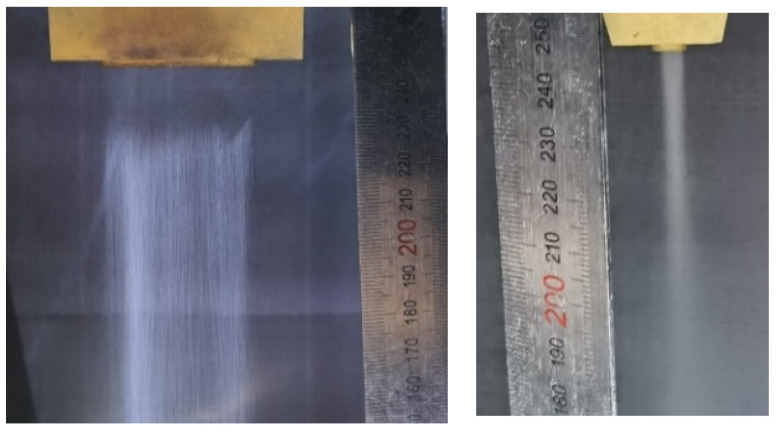
Diagram of powder feeding.

When the powder feed rate is 43.2 g/min, the powder carrier gas rate is 15 L/min, and the collimation gas pressure is 0.1 MPa; the effect of the powder feeding from the nozzle is shown in Figure 19. It shows that two sides of the powder beam conveyed by the powder nozzle have good bunching, a small divergence angle, and uniform distribution, which can meet the expected design requirements.

**Figure 20:**
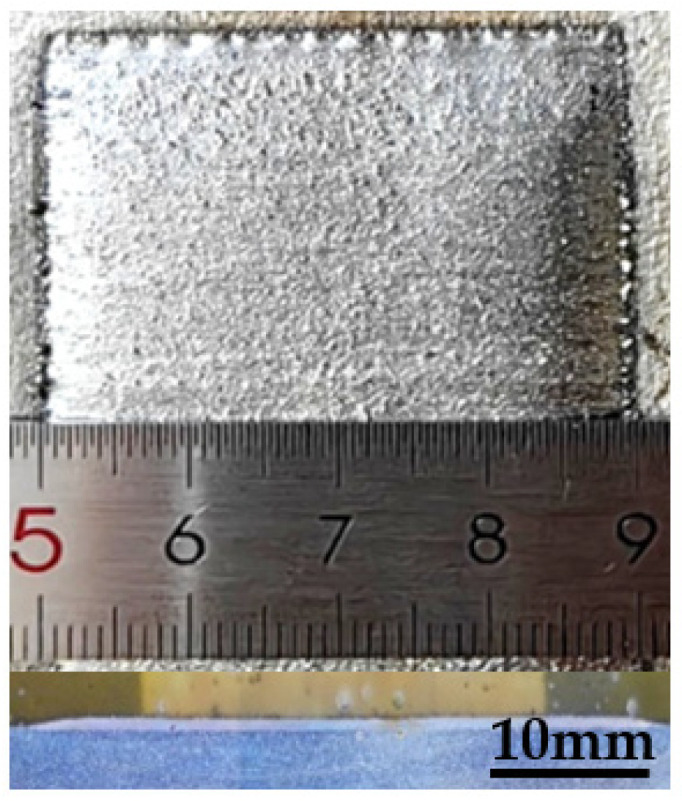
Profile and cross-section diagram of single cladding layer.

A laser wide-band cladding experiment was carried out on 304 stainless steel plates with a RFL-C6000 multi-module continuous fiber laser (Raycus, Wuhan, China), KUKA KR 60HA robot system (KUKA ROBOT, Augsburg, Germany), and GTV PF 2/2 powder feeder. In the process, the laser power was 6 kW, scanning speed was 4 mm/s, and other parameters were the same as before. The result is shown in Figure 20. It can be observed that the width of the cladding layer is 39.96 mm, and the surface is relatively flat, which meets the design requirements.

## 5. Conclusions

1. Utilizing the principles of the inside-beam powder-feeding technology, a novel powder-feeding nozzle for wide-band laser cladding was developed, incorporating a multi-channel powder flow shaping method.
2. A powder distribution evaluation method was established, and through the use of the FLUENT software, the structural parameters of the inside-beam powder-feeding nozzle were selected, including eight powder delivery channels, and the transverse and longitudinal collimating gas exit dimensions were 0.5 mm and 1 mm, respectively.
3. Under varying carrier gas flow rates, the powder concentration distribution exhibited characteristics similar to a Gaussian distribution. As the carrier gas flow rate increased, the powder divergence angle slightly increased, expanding the distribution range. However, within the effective range, the distribution became more uniform, enhancing the stability of the powder transportation. Increasing the powder feed rate resulted in larger divergence angles, while increasing the collimating gas flow rate led to a reduction. Both parameters had a minimal impact on the powder concentration distribution.
4. Using the designed powder-feeding nozzle, a cladding layer with a width of 39.96 mm was achieved at a distance of 40 mm below the nozzle outlet. The convergence of powder in both the length and width directions was excellent, effectively meeting the requirements of wide-band laser cladding.

## Data Availability

Data are contained within the article.
